# Outcome in adult patients with severe TBI, using a management protocol allowing a cerebral perfusion pressure ≤ 60 mmHg – a retrospective study over 10 years

**DOI:** 10.1007/s00701-025-06701-6

**Published:** 2025-10-23

**Authors:** Linus Réen, Anna Radman, Edward Visse, David Cederberg, Niklas Marklund, Peter Siesjö

**Affiliations:** 1 Department of Clinical Sciences Lund, Neurosurgery, Department of Clinical Sciences, Lund University, Lund, Sweden; 2 Department of Neurosurgery, Skane University Hospital, Lund, Sweden

**Keywords:** Traumatic brain injury, Intracranial pressure, Cerebral perfusion pressure, Lund Concept, Neurointensive care, Glasgow Outcome Scale – extended (GOS-E)

## Abstract

**Background:**

Current guidelines for the treatment of severe TBI recommend maintaining a cerebral perfusion pressure (CPP) at 60–70 mmHg. In our institution, as well as others, an alternative algorithm—originally named the Lund concept—has been used. This treatment algorithm employs metoprolol and clonidine to limit CPP, accepting levels below 60 mmHg, with the aim of reducing cerebral edema. Previous reports on this algorithm have shown promising outcome in severe TBI cohorts when compared with many contemporary practices; however, no population-based studies have been conducted to validate these findings.

Research Question:

What is the outcome in adult severe TBI using the Lund Concept algorithm in a population-based cohort and how are CPP levels lower than 60 mmHg tolerated?

**Methods:**

The study included 135 evaluable adult patients out of 171 admitted with severe TBI over a ten-year period in the southern Swedish healthcare region. Baseline data, intracranial pressure (ICP), CPP, treatment duration, surgical interventions, and administered drugs were correlated to the Glasgow Outcome Scale Extended (GOSE).

**Results:**

The 30-day and 6-month mortality rates were 16% and 20%, respectively. A favorable outcome (GOSE 5–8) was achieved in 48% of patients. Only increasing age was associated with inferior outcomes.

**Conclusion:**

The use of a management protocol accepting lower CPP levels than those recommended in existing guidelines was generally well tolerated with outcome in line with comparable reports.

**Supplementary information:**

The online version contains supplementary material available at 10.1007/s00701-025-06701-6.

## Introduction

Severe traumatic brain injury (TBI) remains one of the leading causes of morbidity and mortality in adults despite modern prevention [40] and advances in neurocritical care [24, 30, 39]. Worldwide, TBI is referred to as a silent epidemic with increasing incidence [3, 25, 31]. The surge of motor vehicles, and a lack of safety laws as well as insufficient preventive measures have led to a rise of TBI in low- and middle-income countries, whereas falls in elderly patients are becoming the predominant cause of injury in high-income countries [7, 39, 53]. Country-based estimates demonstrate great variations in fatal outcome after severe TBI. However, these numbers are uncertain as most studies are not population-based, e.g. does not report all cases from a defined population [11, 33, 41, 70] and often mix moderate and severe TBIs [49, 50, 71].

At time of TBI, there is a primary brain injury that occurs as an immediate consequence of the trauma impact, while secondary brain injury is a process that occurs within hours to days thereafter. Secondary brain injury encompasses events such as vasogenic and cytotoxic edema, hypoxia, sterile inflammation, seizures, and excitotoxicity that ultimately lead to the loss of neuronal and glial cells. Brain edema commonly results in increased intracranial pressure (ICP) that is a critical cause of secondary brain injury following TBI [7, 51, 61, 68]. Modern neurocritical care (NCC) is based on the assumption that prevention, of secondary brain injury by monitoring and subsequent treatment improves outcome following TBI. The cornerstone of current TBI treatment is primarily the monitoring of ICP, cerebral perfusion pressure (CPP) and computations of these, guiding interventions such as pharmacological therapy and surgical evacuation of expansive mass lesions including decompressive craniectomy [8, 21]. The complexity and resource demands of this multimodality therapy requires specialized neurointensive care units (Neuro-ICU:s)[7, 37]. Although there is relative general consensus of the basic principles of TBI management, various treatment protocols exist and so far, none of those have been proven to be superior to any other. Recent studies and guidelines recommend treatment of ICP > 20–25 mmHg [1, 8, 10, 57] and maintaining a CPP between 60–70 mmHg [8, 57]. However, optimal thresholds for CPP have been proposed to be patient-specific and time-dependent [2, 28]. The Lund concept (LC) [19, 27, 33] was originally presented in the 1990 s as a method to reduce vasogenic oedema by controlling hydrostatic and osmotic forces in order to maintain fluid volume in the intravascular compartment and minimize transcapillary fluid transport. Specifically, in order to reduce cerebral edema, LC allowed CPP to be lower than recommended by contemporary protocols. To maintain a normovolemic fluid balance and a high hemoglobin concentration (110 g/l), leukocyte-depleted blood transfusions as well as a liberal use of albumin (20% solution) were used. The rationale for using antihypertensive drugs and albumin as the main plasma volume expander was to reduce hydrostatic pressure and restore the plasma oncotic pressure, thereby minimizing transcapillary filtration and lowering ICP. A key component of the protocol is the combined use of clonidine and metoprolol, along with the avoidance of vasopressors, to reduce adrenergic stress and lower arterial blood pressure. The treatment protocol does not include wake-up tests and patients remain sedated with midazolam and fentanyl until ICP stabilization is achieved. In order to keep ICP < 20 mmHg, CPP levels < 60 mmHg can be tolerated. In cases of treatment-refractory ICP > 20 mmHg, management options include low-dose barbiturate administration, ventricular drainage or, as a last resort, decompressive hemicraniectomy [4] (Suppl. Figure 1). The treatment protocol has been much debated [46] but in recent years, guidelines for treatment of TBI and the LC are more similar, especially the recommended thresholds for CPP have decreased in current guidelines. Initial reports of outcome after treatment with LC indicated reduced mortality and morbidity when compared to historical data from the same institutions, but these results may suffer from possible selection biases [19, 45, 60]. To date, no population-based analysis of long-term outcome and how different factors and components of the LC contribute to outcome has been presented.

Therefore, the aim of this study was to evaluate outcome in patients with severe TBI treated according to the LC, and to identify important prognostic factors. We also hypothesized that the use of the specific algorithm encompassing the acceptance of CPP values < 60 mmHg would be well tolerated in patients with severe TBI.

## Material and methods

**Fig. 1:**
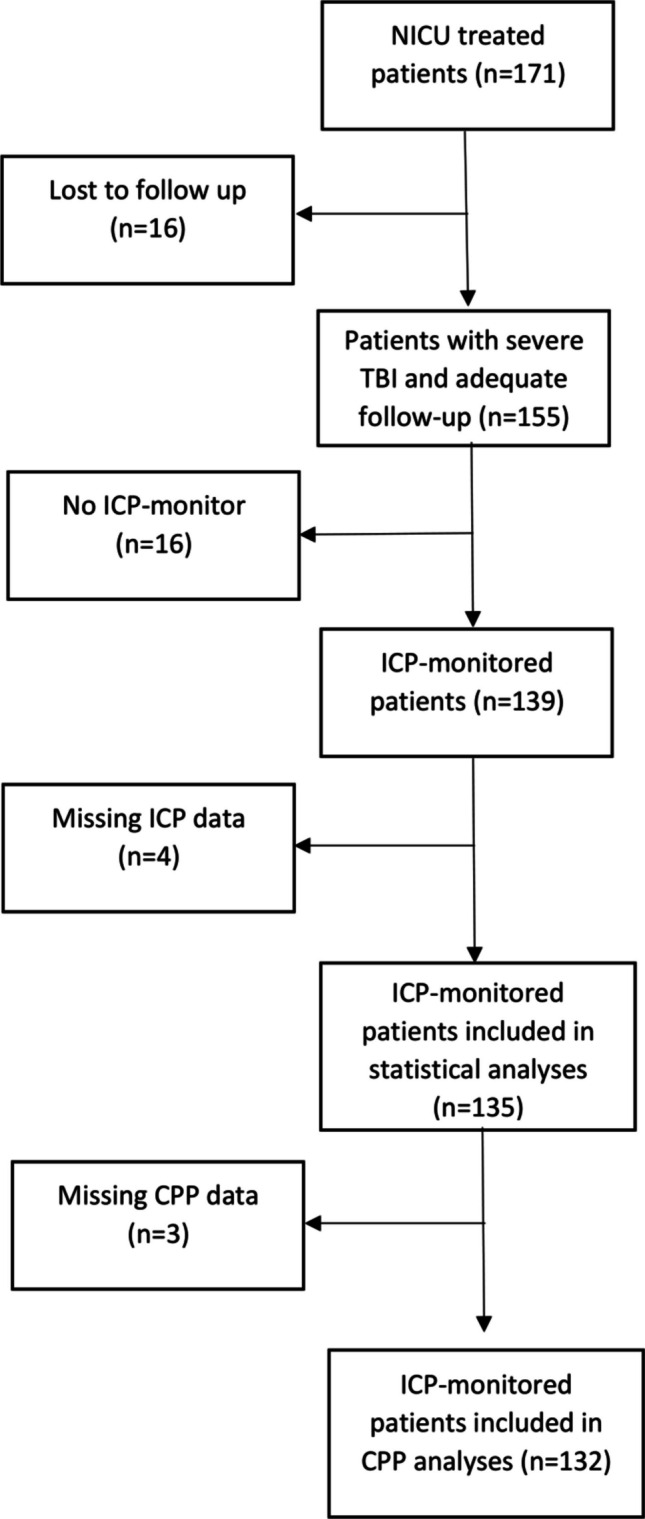
Flowchart of all patients

In the present study, we performed a retrospective analysis of adult patients (≥ 18 years old) with severe TBI, treated at the department of neurosurgery in Lund, Skane University Hospital 2007–2016. As the treatment protocol at the Neuro-ICU, Skane University Hospital, was updated in 2017—among other changes allowing more liberal use of vasopressors and introducing new reference points for ICP and mean arterial pressure (MAP)—only patients treated before 2017 were included to avoid introducing possible bias due to treatment variations within the cohort. Skane University Hospital is the regional tertiary trauma center with exclusive responsibility for the management of severe TBI in the southern region of Sweden, with a mean referral population of 1,500,000 adult inhabitants during the study period, 2007–2017. All adult severe TBI patients, except those where treatment is deemed futile based on clinical and radiological information, are transferred to the NEURO-ICU at Skane University Hospital. Data was collected from medical record systems, including the monitoring system (Intellispace Critical Care and Anesthesia [ICCA] system; Philips, Eindhoven, Netherlands) at the Neuro-ICU. Patients were identified by the International Classification of Diseases (ICD) 10 system (S061-S069) and keywords for TBI used by ICCA. Inclusion criteria were age ≥ 18 years at admission and severe TBI (GCS < 9 following trauma) or GCS ≥ 9 but rapidly deteriorating to GCS < 9 within 24 h. Information regarding type of injury, operations, pharmacological treatment, and physiological variables was collected from medical charts. Radiological features, including Marshall classification [42], were collected from the hospital´s radiology software (Sectra IDS7, Linköping, Sweden). ICP was measured either by an intraventricular catheter, with the highest level of the subarachnoid space as reference point, or an intraparenchymal sensor (Codman®, Integra, Princeton, USA). Values were collected hourly until removal of ICP monitoring devices. Normal ICP values were defined as ICP < 20 mmHg and duration of time with ICP < 15 mmHg, 15–20 mmHg, 21–30 mmHg, and > 30 mmHg were analyzed against outcome. The cerebral perfusion pressure (CPP) was calculated using the mean arterial pressure (MAP), measured by an arterial line at the level of the right atrium and ICP recorded from an intraventricular or intraparenchymal catheter. Optimal CPP for adults with severe TBI is yet to be defined, however various thresholds and upper limits have been suggested [8, 20, 32]. In the present study, duration of time with CPP < 40 mmHg, < 50 mmHg, 50–60 mmHg, and > 60 mmHg were analyzed against outcome. Absolute values of ICP and CPP were defined as the total number of values, whereas relative values were defined as the percentage of values above, below, or within a certain threshold (e.g., CPP > 60 mmHg or CPP 51–60 mmHg). Patient outcomes were estimated using the Glasgow Outcome Scale-Extended (GOSE) [69]-based questionnaires completed by patients and/or next of kin or via structured interviews conducted by study physicians using the same questionnaire. In cases where neither questionnaires were completed nor structured interviews were feasible, outcomes were estimated from medical records. Outcome was defined as good recovery (GOSE 7–8), moderate disability (GOSE 5–6), severe disability (GOSE 3–4), vegetative (GOSE 2) and dead (GOSE 1). Median follow up time was 8 years post-injury (range 2–11 years). Outcomes were assessed in terms of 30-day mortality, 6-month mortality, favorable outcome (GOSE 5–8), and unfavorable outcome (GOSE 1–4). A total of 171 patients treated for severe TBI between 2007 and 2016 at the Neuro-ICU, Skåne University Hospital, Lund, Sweden, were identified, corresponding to an incidence of 1.3 per 100 000 population for patients with severe TBI admitted to the tertiary Neuro-ICU. Predicted outcomes generated using the IMPACT core model [70, 71] were compared with the actual patient outcomes. Patients without available ICP data and/or those lost to follow-up were excluded from all analyses (Fig. 1).

### Statistical analysis

Chi square test was used for categorical data. For the analysis of the effect by different factors on GOSE the following dichotomies were assessed by logistic regression (*glm, binary* in R); 8 versus 1–7 and 5–8 versus 1–4. A p-value ≤ 0.05 was considered statistically significant. To assess whether statistics designated for ordinal stepwise data would give more information, an ordinal regression test, cumulative link models (*clm* in R), was used and compared to *glm* of dichotomized GOSE outcomes. Statistically significant values from the ordinal regression test were also compared to the corresponding dichotomies in the *glm* analysis. Bonferroni correction was applied to account for multiple testing. Parametric data are presented as mean +—standard deviation (SD). Nonparametric data are presented as median and interquartile range (IQR). The odds ratio (OR) is presented with a 95% confidence interval (CI). Multivariable GLM (Generalized Linear Models) was conducted to assess the association between clinical variables and outcome. The model included the following covariates: Age, GCS, pupillary reactivity, Marshall classification, ICP > 20 mmHg, and absolute values of CPP 51–60 mmHg. Variables were selected based on results from the univariate analysis and clinical relevance. To assess collinearity, pairwise Pearson correlation coefficients were calculated, with a threshold of ≥ 0.8 set to indicate high collinearity. All computations were run with the free statistical software R (https://www.r-project.org).

## Ethics

Ethical approval was granted by local ethics committee (Regionala Etikprövningsnämnden Box 133/HS 12, 221 00 Lund). Reference number: 2017/469. The authors confirm that this study was conducted in accordance with the declaration of Helsinki.

## Results

### Patients and radiological appearance

Patient characteristics are shown in Table 1. The initial CT scan was classified as Marshall 5 or 6 in the majority of patients (62%). There was a high male predominance (98 males vs 37 females; 73%). Median age was 49 (range 18–74) years old and median initial GCS was 7 (range 3–15). Pre-injury Co-morbidities were identified in 75 (56%) patients. The most prevalent were hypertension (17%), followed by diabetes (9%), alcohol abuse (8%) and depression (6%). Data on pupil size and reactivity was available in 117 patients (87%). Pupillary abnormalities were observed in 39 patients (33%), of whom 21 (18%) had a unilateral fixed and dilated pupil, and 10 (9%) had bilateral fixed and dilated pupils. The predominant radiological diagnosis was cerebral contusions (83%), followed by subdural hematomas (58%). A majority (73%) of patients suffered from multiple intracranial injuries, and 30 patients (22%) had various degrees of extracranial injuries. Sixteen patients (9%) were lost to follow up and were excluded from all analyses.

**Table 1:** Patient characteristics. Clinical and radiological features of all admitted patients presented as absolute values (n) and relative values (%). EDH – Epidural Hematoma, GCS – Glasgow Coma Scale, SDH – Subdural Hematoma, TSAH – Traumatic Subarachnoid Hemorrhage

	*n*	%
Patients	135	100
Age (median)	49	-
Male	98	73
Female	37	27
GCS 3–8	92	68
GCS 9–13	31	23
GCS 14–15	12	9
Marshall 1	2	1
Marshall 2	38	28
Marshall 3	11	8
Marshall 5	83	61
Marshall 6	1	< 1
EDH	18	13
SDH	69	51
TSAH	51	38
Contusion	98	73
Pupil dilatation ^a^	31	27
-bilateral	10	9
-unilateral	21	18
Comorbidities	75	54
-hypertension	23	17
-alcohol abuse	15	11
-diabetes	12	9
-depression	9	7

## NEURO-ICU treatment

ICP monitoring was performed in all included patients. Surgical evacuation of mass lesions was performed in 94 patients (70%), decompressive craniectomy (DC) in 18 patients (13%), and 41 patients (30%) were managed with ICP-monitoring alone. Median NEURO-ICU stay was 224 h (range 18–1055 h).

## NEURO-ICU drugs

A majority of patients (87%) were initially sedated with propofol; however, midazolam was used in 74% of patients, either initially or later during the course of treatment. Metoprolol and clonidine were administrated in 67% and 76% of patients, respectively. Pentothal was used in 19% of patients (*n* = 26) (Table 2).

**Table 2:** Summary of surgical and medical treatment. Absolute values (*n*) and relative values (%) are presented respectively. Medical treatment is shown as median values of total doses. DC – Decompressive Hemicraniectomy, ICP – Intracranial Pressure

	*n*	%
Decompressive surgery and ICP-monitoring	94	70
Only ICP-monitor	41	30
DC	18	13
Propofol	118	87
- median dose (mg)	3280	-
Midazolam	100	74
-median dose (mg)	1890	-
Metoprolol	91	67
-median dose (mg)	126	-
Clonidine	103	76
-median dose (ug)	2250	-
Pentothal	26	19
-median dose (mg)	8175	-

## ICP and CPP

From 135 ICP-monitored patients with accurate data, a total of 23,120 ICP hourly values were collected, and a total of 22,205 CPP-values could be collected from 132 ICP-monitored patients. Only 2% of ICP values exceeded 20 mmHg while 75% were below 15 mmHg. CPP was kept > 50 mmHg, 95% of time and < 1% of CPP values were < 40 mmHg. In total, 22% of CPP values were < 60 mmHg. (Table 3).

**Table 3:** A summary of all hourly collected ICP- and CPP values. Absolute (*n*) and relative (%) values for different cutoffs are shown. CPP – Cerebral Perfusion Pressure, ICP – Intracranial Pressure

				*n*	%
ICP (all values)				23,120	100
< 15 mmHg				17,301	75
15–20 mmHg				5375	23
21–30 mmHg				351	1
> 30 mmHg				93	< 1
CPP (all values)				22,205	100
< 40 mmHg				122	< 1
40–50 mmHg				1027	5
51–60 mmHg				3820	17
> 60 mmHg				17,236	78

## Outcome

### Outcome results

Of the 155 patients who had full GOSE follow up, outcomes were obtained through questionnaires and/or structured interviews in 121 patients, and from medical records in 34 patients. Median follow up time was 8 years post-injury (range 2–11 years).

## Patient demographics and outcome

**Fig. 2:**
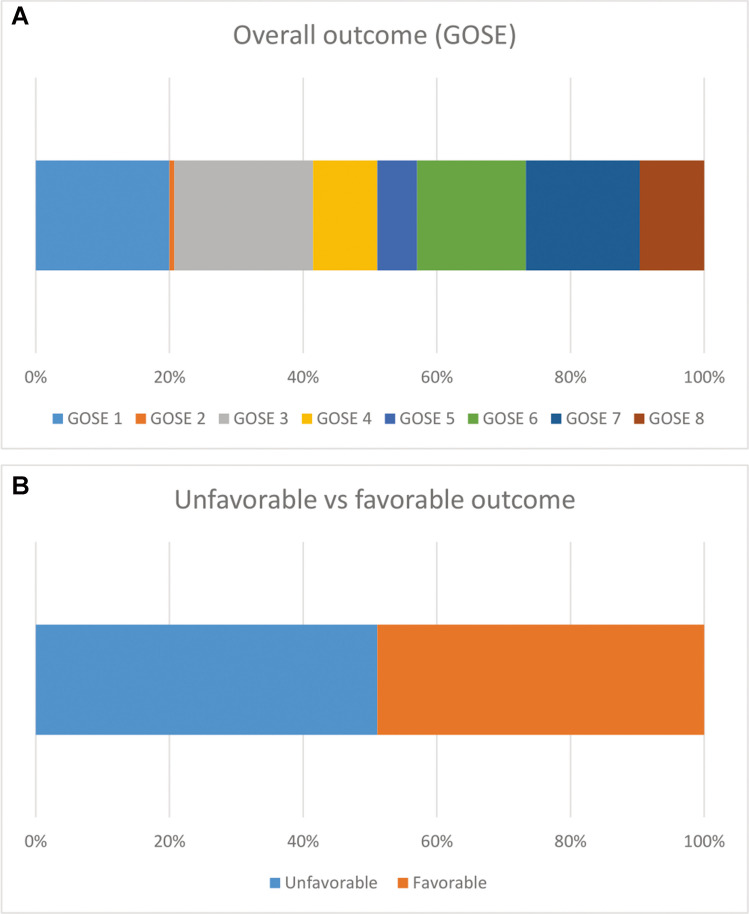
Outcome and 6-month mortality shown as full 8 level GOSE scale **A**, unfavorable/favorable **B**. GOSE – Glasgow Outcome Scale Extended

Clinical parameters and physiological variables are stratified by outcome in Table 4. Median GOSE was 4. The NEURO-ICU mortality was 7%, the 30-day mortality was 16% and 6-month mortality 20%. A favorable outcome, defined as GOSE 5–8, was achieved in 48% of patients and 52% had an unfavorable outcome (GOSE 1–4) (Fig. 2). Good outcome (GOSE 7–8) was achieved in 27% of patients. The predicted mortality and favorable outcome rates according to the IMPACT core model were 29% and 49%, respectively. There was a statistically significant correlation between high age and mortality (*p* < 0.05).

**Table 4:** Clinical parameters and ICP/CPP, stratified by outcome. Patients with favorable and unfavorable outcome are presented in A. Deceased and alive patients are presented in B. Significant *p*-values are shown in bold. CPP – Cerebral Perfusion Pressure, CI – Confidence Interval GCS – Glasgow Coma Scale, IQR – Interquartile Range, ICP – Intracranial Pressure, OR – Odds Ratio. ^a^ Information regarding pupillary status was missing in 18 patients (13%)

A	Favorable	Unfavorable	OR (95% CI)	*p*-value
Age (median (IQR))	38 (33)	57 (26.5)	0.960 (0.939–0.979)	**0.0000853**
GCS (median (IQR))	8 (3)	6 (5)	1.056 (0.961–1.162)	0.261
Unilateral pupil dilatation (*n* (%))^a^	10 (18%)	13 (21%)	N/A	0.488
Bilateral pupil dilatation (*n* (%))	8 (14%)	5 (8%)	N/A	0.488
Unilateral pupil light respons (*n* (%))	9 (16%)	10 (16%)	N/A	1.000
No pupil light respons (*n* (%))	7 (13%)	8 (13%)	N/A	1.000
Marshall (median (IQR))	5 (3)	5 (3)	0.983 (0.773–1.251)	0.888
Relative ICP > 20 mmHg (median (IQR))	0.026 (0.073)	0.020 (0.091)	0.230 (0.005–7.624)	0.419
Absolute ICP > 20 mmHg (median (IQR))	3 (15.5)	3.5 (19.5)	1.003 (0.989–1.018)	0.657
Relative CPP > 60 mmHg (median (IQR))	0.824 (0.303)	0.853 (0.270)	0.305 (0.058–1.433)	0.142
Absolute CPP > 60 mmHg (median (IQR))	89 (116)	120 (128)	1.000 (0.996–1.003)	0.908
Relative CPP 51–60 mmHg (median (IQR))	0.134 (0.252)	0.106 (0.178)	3.294 (0.255–44.718)	0.363
Absolute CPP 51–60 mmHg (median (IQR))	23.5 (40.5)	16.5 (29.5)	1.007 (0.996–1.019)	0.233
Relative CPP < 50 mmHg (median (IQR))	0.0194(0.0639)	0.0195(0.0639)	0.458(0.0076—15.535)	0.665
Absolute CPP < 50 mmHg (median (IQR))	3.5(11)	3(8)	1.0019(0.9771—1.0275)	0.878
B	Deceased (6 months)	Alive	OR (96 % CI)	*p*-value﻿
Age (median (IQR))	63 (13)	43 (35)	1.060 (1.030–1.098)	**0.000278**
GCS (median (IQR))	6 (9)	8 (4)	0.984 (0.870–1.104)	0.784
Unilateral pupil dilatation (*n* (%))	3 (12%)	20 (22%)	N/A	0.464
Bilateral pupil dilatation (*n* (%))	0 (0%)	13 (14%)	N/A	0.464
Unilateral pupil light respons (*n* (%))	4 (24%)	15 (15%)	N/A	0.491
No pupil light respons (*n* (%))	1 (6%)	14 (17%)	N/A	0.491
Marshall (median (IQR))	5 (3)	5 (3)	0.968 (0.722–1.315)	0.831
Relative ICP > 20 mmHg (median (IQR))	0.020 (0.052)	0.023 (0.085)	1.210 (0.010–67.033)	0.930
Absolute ICP > 20 mmHg (median (IQR))	3 (8.5)	3.5 (20.2)	0.978 (0.941–1.003)	0.172
Relative CPP > 60 mmHg (median (IQR))	0.877 (0.225)	0.828 (0.279)	3.163 (0.424–35.376)	0.302
Absolute CPP > 60 mmHg (median (IQR))	68 (142)	117 (118)	0.999 (0.994–1.003)	0.582
Relative CPP 51–60 mmHg (median (IQR))	0.098 (0.084)	0.134 (0.232)	0.046 (0.001–1.406)	0.094
Absolute CPP 51–60 mmHg (median (IQR))	8 (14.5)	23 (38.5)	0.972 (0.947–0.992)	**0.016**
Relative CPP < 50 mmHg (median (IQR))	0.00637(0.04)	0.0253(0.0662)	5.538(0.0931—294.842)	0.358
Absolute CPP < 50 mmHg (median (IQR))	1(4)	4(10.5)	0.981(0.9358—1.0141)	0.321

## ICP and outcome

In the logistic regression analysis, both absolute and relative ICP values of 15–20 mmHg were significantly lower in deceased patients when compared to those alive at the 6-month follow-up. However, this could not be confirmed after applying Bonferroni correction and no statistical significance could be found between ICP and outcome in the ordinal regression analysis.

## CPP and outcome

**Fig. 3:**
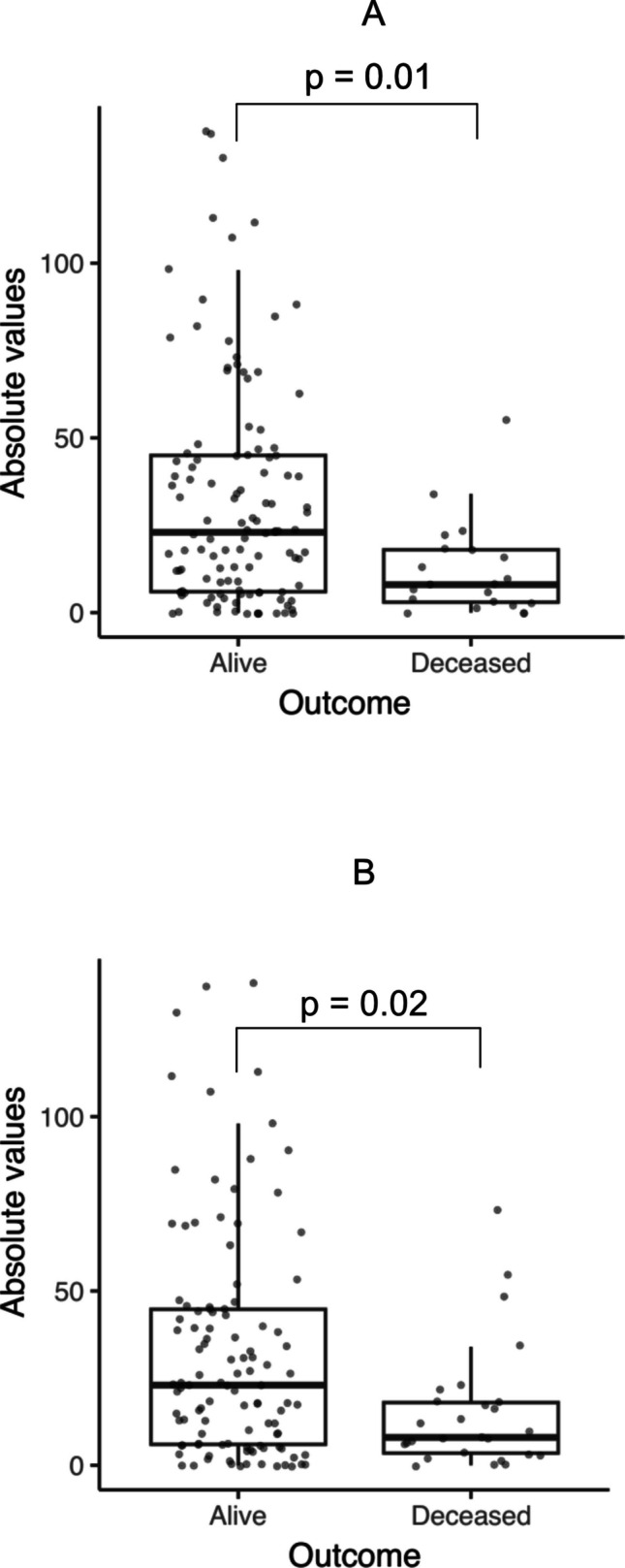
Boxplots illustrate absolute values (hours) of CPP 51–60 mmHg in alive and deceased patients. **A** compare 30-day mortality and **B** compare 6-month mortality. The difference remained significant after applying Bonferroni correction for multiple testing but not in the multivariate analysis. Dots represent individual patients. CPP – Cerebral Perfusion Pressure

Mean CPP monitoring times did not differ significantly between alive and deceased patients (174 +—119 h vs 141 + −121 h; p = 0.1). Absolute CPP values of 51–60 mmHg were significantly lower in deceased patients when compared to those alive in the logistic regression analysis (Fig. 3). However, no statistically significant difference was found in the ordinal regression analysis. The difference remained significant after correction for multiple testing but was not significant in the multivariate analysis (Table 5).

**Table 5:** Results from the multivariate analysis are presented with odds ratios and *p*-values. Age consistently showed statistical significance in all outcome comparisons. CPP – Cerebral Perfusion Pressure, CI – Confidence Interval, GCS – Glasgow Coma Scale, GOSE – Glasgow Outcome Scale Extended, ICP – Intracranial Pressure, OR – Odds Ratio

	OR (95% CI)							
	Favorable vs unfavorable outcome		GOSE 87 vs GOSE 1–6		30-day mortality		6-month mortality	
Age	0.988 (0.983—0.993)		0.990 (0.986—0.995)		1.006 (1.002—1.010)		1.008 (1.004—1.012)	
GCS	1.028 (1.005—1.052)		1.013 (0.992—1.034)		0.994 (0.978—1.012)		0.989 (0.971—1.008)	
Marshall Classification	1.055 (0.967—1.091)		1.026 (0.9715—1.083)		0.980 (0.937—1.024)		0.969 (0.923—1.017)	
ICP > 20 mmHg	1.000 (0.996—1.004)		1.002 (0.998—1.005)		1.000 (0.998—1.003)		1.000 (0.996—1.003)	
CPP 51–60 mmHg (Absolute values)	1.000 (0.996—1.003)		0.998 (0.995—1.001)		0.998 (0.996—1.001)		0.999 (0.996—1.002)	
		*p*-values						
		Favorable vs unfavorable outcome		GOSE 87 vs GOSE 1–6		30-day mortality		6-month mortality
Age		** < 0.001**		** < 0.001**		**0.001**		** < 0.001**
GCS		**0.018**		0.217		0.522		0.263
Marshall Classification		0.363		0.361		0.367		0.209
ICP > 20 mmHg		0.957		0.378		0.736		0.786
CPP 51–60 mmHg (Absolute values)		0.804		0.271		0.139		0.401

## Clonidine/Metoprolol and outcome

Patients treated with clonidine were significantly younger but did not differ significantly in Glasgow Coma Scale (GCS) scores or Marshall classification compared to those who did not receive clonidine (Table 6). However, they had a statistically significant lower mortality (*p* = 0.01). No association was found between metoprolol treatment and patient outcome.

**Table 6:** Age, GCS, and Marshall classification in patients receiving clonidine (*n* = 103) compared to those not receiving clonidine (*n* = 32). Significant *p*-values are shown in bold. GCS – Glasgow Coma Scale, IQR – Interquartile Range

	Clonidine		
	Yes	No	p-value
AGE (median (IQR)	40 (24–57)	65 (57–70)	** < 0.00001**
GCS (median (IQR)	7 (5–9)	8 (5–13)	0.19
Marshall Classification (median (IQR)	4 (2–4)	4 (2–4)	0.25

## Discussion

In this descriptive study—the largest patient series to date evaluating the LC for the treatment of severe TBI—we report a 6-month mortality rate of 20% and a favorable outcome of 48%, which is comparable to recent outcome studies in severe TBI[9, 17, 29, 35, 38, 47, 67]. CPP values between 51 and 60 encompassing 78% of all CPP values < 60 mm Hg did not significantly have worse or better outcome than those > 60 mmHg. As one aim of the study was to investigate whether CPP values < 60 mmHg adversely affect outcomes, it would have been plausible to analyse both the absolute and relative time spent below 60 mmHg compared to time spent above this threshold. However, since 77% of the time with CPP < 60 mmHg was within the 51–60 mmHg range and only 23% below 50 mmHg, such an analysis was deemed unlikely to provide additional relevant information and was therefore not performed.

The low incidence of severe TBI observed in the present study most likely reflects the increased implementation of safety measures in traffic and workplaces over recent decades. Nevertheless, our findings are consistent with those of a recent Nordic population-based study, which reported an incidence of 2.9 per 100 000 inhabitants [67].

In earlier studies evaluating the LC, a mortality rate of 3–20% of adult patients with severe TBI was observed, as well as a favorable outcome of 52–82% [33]. However, it is difficult to compare these previous data with those from the present study, since important prognostic indicators, such as the Marshall classification and pupil status, were often missing [18, 44]. Previous studies also involved smaller, selected, non-population-based cohorts of predominately younger patients [18, 44, 45, 48, 60]. As demonstrated by the present and many previous studies, age is a significant negative predictor of outcome in severe TBI [15, 23, 24], which may partially account for the notably low morbidity and mortality observed in prior studies evaluating the LC [18, 45].

We previously reported the long-term outcome in children <18 years with severe TBI treated according to the LC [56]. When compared to these results in the pediatric population, mortality is doubled, and favorable outcome is reduced from 75 to 48% in adult patients treated with the same algorithm. While these results emphasize that children <18 years have a more favorable prognosis than adults after severe TBI, we cannot exclude that the tolerance and efficacy of the LC is better in the pediatric population.

Mortality and outcome in severe TBI are generally lower in randomized trials (RCT) than from population-based studies, which could be explained by selection bias in RCTs. The present mortality and outcome rates are similar to recent RCTs, and in relevant population-based studies of severe TBI from countries with a high Human Development Index (HDI), where mortality ranges from 25–51% and favorable outcome between 34–51% respectively have been reported [13, 17, 29, 49, 50, 52, 64, 67]. In the present study all patients admitted to the tertiary NEURO-ICU were included and thus the population also encompassed patients in GCS 3 and fixed dilated pupils who sometimes are excluded in RCTs.

A fundamental aspect of the LC involves the administration of metoprolol and clonidine to reduce the hydrostatic pressure with the intent of minimizing capillary fluid leakage through an impaired blood–brain barrier (BBB) [4, 27]. However, the use of antihypertensive agents is sometimes discouraged due to the risk of unacceptably low CPP values (< 50 mmHg), which may partially explain why not all patients received metoprolol and clonidine (67% and 76% respectively) in this study. The finding that patients receiving clonidine had significantly lower mortality despite similar injury severity compared to those not receiving clonidine should be interpreted with caution, as the clonidine group was significantly younger. However, we cannot rule out that the choice of not using clonidine and/or metoprolol was influenced by factors other than avoidance of low blood pressure and/or CPP values. Current TBI-guidelines [6, 8] suggest that CPP should be kept at a minimum of 60–70 mmHg which is a lower goal than in previous guidelines. The clinical benefit of the commonly used inotropic drugs in the management of severe TBI remains a subject of debate [36]. However, liberal use of vasopressors has been associated with a five-fold increased risk of acute respiratory distress syndrome (ARDS) [12]. A basic assumption for the rationale of LC was that elevated CPP could exacerbate cerebral edema [4, 18, 27] due to a disruption of the BBB resulting in impaired autoregulation [27] observed in 49–87% of patients with severe TBI [55]. Since the introduction of the pressure reactivity index (PrX) [14] and optimal CPP (CPPopt) [59] there has been a growing interest in individualized CPP-management [26, 63, 65, 72]. Even though this is theoretically appealing, to date there is still no gold standard for estimating cerebral autoregulation [16], and there is conflicting evidence regarding the clinical benefits of CPPopt-guided treatment [34, 58, 72]. Moreover, CPPopt has only been prospectively evaluated by comparing CPPopt-guided treatment to treatment according to current guidelines (CPP 60–70 mmHg) [63] and not compared to lower CPP thresholds. The present study does not challenge the notion that CPP values lower than guideline recommendations may be tolerated without adverse impact on outcome. Our findings closely align with a previous report including patients in poor clinical condition, such as dilated pupils and/or low GCS scores[20]. Thus, the severely injured adult brain may be sensitive to both low and high CPP values suggesting that a narrow target range, rather than a lower limit, is warranted. The absence of a correlation between low CPP values and outcome is most likely explained by the high rate of CPP control achieved (95% of CPP > 50 mmHg). It is also worth noting that, although a CPP of 51–60 mmHg is accepted by the LC, active reduction of CPP in an otherwise stable patient is not standard clinical practice. This is reflected, in part, by the finding that only 17% of recorded CPP values fell within the 51–60 mmHg range, whereas 78% exceeded 60 mmHg.

The low percentage of elevated ICP in our study may argue that LC is effective in achieving ICP control in a majority of patients. One of the core hypotheses of the Lund concept is that early, active reduction of CPP decreases capillary fluid extravasation and thereby reduces ICP in patients with severe TBI. However, the reason for the high level of ICP control is most likely multifactorial and not solely attributable to the treatment protocol. The relatively low incidence of DC (13%) compared to other studies [29] may also argue for the potential of pharmacologically managing moderately elevated ICP-values.

We acknowledge that there are several limitations to the present study. As with all retrospective studies, there are inherent uncertainties regarding data accuracy, various forms of bias and confounding factors. In addition, the descriptive nature of the study inherently limits any conclusions regarding causality. Data was collected over a relatively long time period, which may have affected the results. ICP and CPP were only recorded every hour, which in conjunction with the stringent control of CPP and ICP may explain the lack of correlation between high ICP/low CPP and outcome. High-frequency data sampling would have been desirable for determining optimal ICP and CPP thresholds in future studies. Moreover, the significantly higher frequence of CPP values between 51–60 mmHg in survivors, as shown in the present study, should be interpreted with much caution, as it may be influenced by confounding factors such as cardiovascular disease, age, secondary insults, autoregulatory status and other variables. As stated in the results, most of these correlations did not remain significant after applying Bonferroni correction or multivariate analyses. Thus, while CPP < 60 mmHg was well tolerated in this cohort, we do not argue that CPP should be maintained < 60 mmHg in all adult severe TBI patients. Severe TBI encompasses a range of diagnoses and optimal CPP ranges in patients with predominantly contusions and TSAH most likely differ from those in patients with extra-axial mass lesions and unstable ICP. As stated previously, only 67% of all patients were treated with metoprolol. Since 1/3 of patients did not adhere to the LC algorithm, outcome data cannot be interpretated with certainty and the LC may not be beneficial for all severe TBI patients.

Another limitation with this study is that data on the use of albumin and blood transfusions were missing. The role of blood transfusions in severe TBI remains unclear with current guidelines offering cautious support for restrictive transfusion protocols [43]; however recent randomized trials suggest that liberal transfusion is not associated with a higher risk of complications and may improve outcome in severe TBI [62, 66]. Given that the LC advocates for the liberal use of transfusions and albumin, it is possible that these factors influenced patient outcome in the present study. The role of albumin in TBI treatment remains debated, with a previous randomized trial reporting an association with poorer outcomes. In contrast, the LC concept advocates the use of 20% albumin—a hyperosmolar solution—in addition to crystalloids, whereas the referenced trial employed exclusive use of 5% albumin—a hypoosmolar solution—making direct comparison difficult.

Although the low rate of elevated ICP may suggest that the LC is effective in achieving ICP control, it may also explain why CPP < 60 mmHg was well tolerated. This does not provide evidence that maintaining CPP < 60 mmHg is beneficial in clinical scenarios involving, for example, vasospasm and/or ICP instability. Another limitation of the present study is that head elevation, a common practice in the management of severe TBI, was not accounted for as the level of head elevation was not recorded in the medical charts. Theoretically, this could alter CPP values, leading to ambiguous results. Furthermore, the diagnosis of severe TBI was based on the initial GCS with further evaluation limited by intubation and sedation. This may have led to inaccurate GCS scoring of patients, potentially skewing the outcome data. Our Neuro-ICU at Skane University Hospital is a referral center for the southern Swedish health region thus admitting all patients considered eligible for the treatment based considering comorbidity, age, and severity of TBI. Although this ascertains a relatively constant selection over time, we cannot rule out selection bias by individual physicians on call. Moreover, variability in the timing of outcome assessments may have introduced bias, thereby compromising the reliability and comparability of the data.

The Marshall Classification remains widely used in recent relevant studies [13, 29, 39, 60] and was used for radiological classification of TBI in the present study. Although several previous studies suggest that alternative radiological classifications may provide a more accurate description of injury severity and improved outcome prediction in severe TBI [5, 22, 54], no universal gold standard currently exists. In the present study, no significant correlation was found between outcome and Marshall Classification in either univariate or multivariate analyses.

## Conclusion

Low morbidity and mortality rates, comparable to those reported in recent outcome studies employing alternative treatment protocols, were observed in adult patients with severe TBI managed according to the LC.

## Supplementary information

Below is the link to the electronic supplementary material.

Supplementary file 1 (TIFF 570 KB)

## Data Availability

The datasets used and/or analysed during the current study are not publicly available due to study participant privacy but are available from the corresponding author on reasonable request.

## Declarations

### Artificial intelligence

The grammar and spelling of this study have, in part, been reviewed using ChatGPT (https://openai.com/index/chatgpt/ChatGPT | OpenAI). The authors have processed the generated text and images and take full responsibility for the content.
